# Contrasting Soil Bacterial Community, Diversity, and Function in Two Forests in China

**DOI:** 10.3389/fmicb.2018.01693

**Published:** 2018-07-31

**Authors:** Hua Wei, Changhui Peng, Bin Yang, Hanxiong Song, Quan Li, Lin Jiang, Gang Wei, Kefeng Wang, Hui Wang, Shirong Liu, Xiaojing Liu, Dexiang Chen, Yide Li, Meng Wang

**Affiliations:** ^1^Center for Ecological Forecasting and Global Change, College of Forestry, Northwest A&F University, Yangling, China; ^2^Medical College, Baoji Vocational Technology College, Baoji, China; ^3^Départment des Sciences Biologiques, Institut des Sciences de l'Environnement, Université du Québec à Montréal, Montreal, QC, Canada; ^4^Research Institute of Forest Ecology, Environment and Protection, Chinese Academy of Forestry, Beijing, China; ^5^Baotianman Natural Reserve Administration, Neixiang, China; ^6^Research Institute of Tropical Forestry, Chinese Academy of Forestry, Guangzhou, China; ^7^State Environmental Protection Key Laboratory of Wetland Ecology and Vegetation Restoration, Institute for Peat and Mire Research, Northeast Normal University, Changchun, China

**Keywords:** bacterial community structure, bacterial diversity, bacterial function, temperate deciduous broad-leaved forest, tropical rainforest, environmental factors

## Abstract

Bacteria are the highest abundant microorganisms in the soil. To investigate bacteria community structures, diversity, and functions, contrasting them in four different seasons all the year round with/within two different forest type soils of China. We analyzed soil bacterial community based on 16S rRNA gene sequencing via Illumina HiSeq platform at a temperate deciduous broad-leaved forest (Baotianman, BTM) and a tropical rainforest (Jianfengling, JFL). We obtained 51,137 operational taxonomic units (OTUs) and classified them into 44 phyla and 556 known genera, 18.2% of which had a relative abundance >1%. The composition in each phylum was similar between the two forest sites. *Proteobacteria* and *Acidobacteria* were the most abundant phyla in the soil samples between the two forest sites. The Shannon index did not significantly differ among the four seasons at BTM or JFL and was higher at BTM than JFL in each season. The bacteria community at both BTM and JFL showed two significant (*P* < 0.05) predicted functions related to carbon cycle (anoxygenic photoautotrophy sulfur oxidizing and anoxygenic photoautotrophy) and three significant (*P* < 0.05) predicted functions related to nitrogen cycle (nitrous denitrificaton, nitrite denitrification, and nitrous oxide denitrification). We provide the basis on how changes in bacterial community composition and diversity leading to differences in carbon and nitrogen cycles at the two forests.

## Introduction

Forests are vital components of the terrestrial ecosystem. In forest ecosystem, soils play an important role in mediating fundamental nutrient and energy flow patterns (Canadell and Raupach, 2008). Soil bacteria are the primary drivers of these ecological processes (Six et al., 2006; Bardgett et al., 2008). Some bacteria are associated with the production or absorption of greenhouse gases such as CO_2_, CH_4_, and N_2_O (Lladó et al., 2017). *Proteobacteria* and *Acidobacteria* are the most phyla in soil bacteria (Roesch et al., 2007; Lauber et al., 2009; Nemergut et al., 2010). *Proteobacteria* predominated in the natural hardwood forest soils, but *Acidobacteria* was the most abundant phylum in the secondary coniferous forest soil communities (Lin et al., 2011).

Environmental factors play a vital role in determining the bacterial community structure of forest soils than geographic dispersal limitation (Xia et al., 2016). It is widely accepted that the structure and diversity of soil bacteria are influenced by soil properties and vegetation types. Soil pH is considered as an important factor in controlling bacterial community structure (Qiu et al., 2014). Other soil characteristics also affect soil bacterial community composition and diversity, such as nutrient availability (Broughton and Gross, 2000; Liu et al., 2010; Naether et al., 2012) and plant diversity (Stephan et al., 2000; Wardle et al., 2004). The distribution of bacterial communities in previous studies found that soil bacterial community was influenced by soil property, climatic, or other characteristics (Cho and Tiedje, 2000; Zhou et al., 2002; Yergeau et al., 2007). The controlling factors varied with different ecosystem types at different spatial scales.

The microbial functions and its related process were determined by microbial communities (Waldrop and Firestone, 2006; Fierer et al., 2013). Microorganisms especially bacteria regulate soil ecosystem function (e.g., nutrient cycling, decomposition of organic matter, soil structure, greenhouse gases) (Rousk and Bengtson, 2014). The study on the biological processes of carbon and nitrogen in soil bacteria is necessary for the investigation of global climate change. Meanwhile, the soil carbon and nitrogen cycle and their associated bacteria are also the key component in the study of climate change. Therefore, the changes of bacteria community structure and diversity are closely related to climate change.

Previous studies have examined the relationships between ecosystem functioning and community structures, but most are limited to grassland and cropland ecosystems (Larsen et al., 2005; Soussana et al., 2012). The forests play a very important role in global carbon cycle, so the forests soil ecology is a vital research field. Bacteria contribute to many essential soil processes such as the carbon and nitrogen cycles. In forest soils, bacteria may respond to climate change actively (Lladó et al., 2017). This response often reflects the specificities of each studied forest ecosystem. High-throughput sequencing can assess the diversity and the role of bacterial communities in the complex forest ecosystem (Staley et al., 2013; Sun et al., 2014). However, it is still unclear what are the major drivers of bacteria abundance and diversity and how do they respond to climate change.

Baotianman Nature Reserve (BTM) and Jianfengling National Natural Reserve (JFL) are located in climatic transitional zones. BTM has the characteristics of the transition between north subtropical and warm temperate zone. JFL has the characteristics of transition from tropical to subtropical. The main soil type is brunisolic soil at Baotianman (Du et al., 2016) and podzolic soil for JFL (Cheng et al., 2007). The degree of habitat heterogeneity is relatively high in the climatic transition zone (ecological transition zone), leading to the increase of biological diversity and richness, particularly sensitive to global climate change. At the same time, global biodiversity research on transition zones attracts more attention of ecologists worldwide (Brooks et al., 2006). So they are the ideal locations to study soil bacterial diversity, composition, and function. The study on the biological processes of carbon and nitrogen in soil bacteria is of great significance for the maintenance of ecosystem services under global climate change.

With different climate and vegetation type, the soil physiochemical properties at BTM and JFL differ all the year round. The soil physiochemical properties (e.g., soil water content, temperature, etc.,) affect the structure and diversity of bacterial community, meanwhile the structure and diversity of bacterial community are significantly related to the bacterial function. Soil microbial diversity decreases with the increase of latitude (Staddon et al., 1998), which indicates that soil microbial diversity at JFL is probably higher than that at BTM. Due to the abundant precipitation, suitable temperature and large plant diversity at JFL, we assume that the soil bacterial community richness and diversity will be greater at JFL than BTM, and the predicted functions related to the carbon and nitrogen cycle regulated by bacteria are stronger at JFL than the BTM. To the best of our knowledge, this study is the first to characterize bacterial community structures and functions in these two transitional forest soils.

The aim of our study were to (1) compare the soil bacterial community structure and diversity in the two forest sites of China; (2) identify the physicochemical of soil which affect bacterial community significantly; (3) examine the relationships between bacterial community structures and their potential functions. Our comparison of the changes in soil bacterial diversity and function between BTM and JFL will advance the understanding of soil carbon and nitrogen cycling and they may be linked with future climate change in forest ecosystems in the transitional climate regions.

## Materials and methods

### Field sites

**Figure 1:**
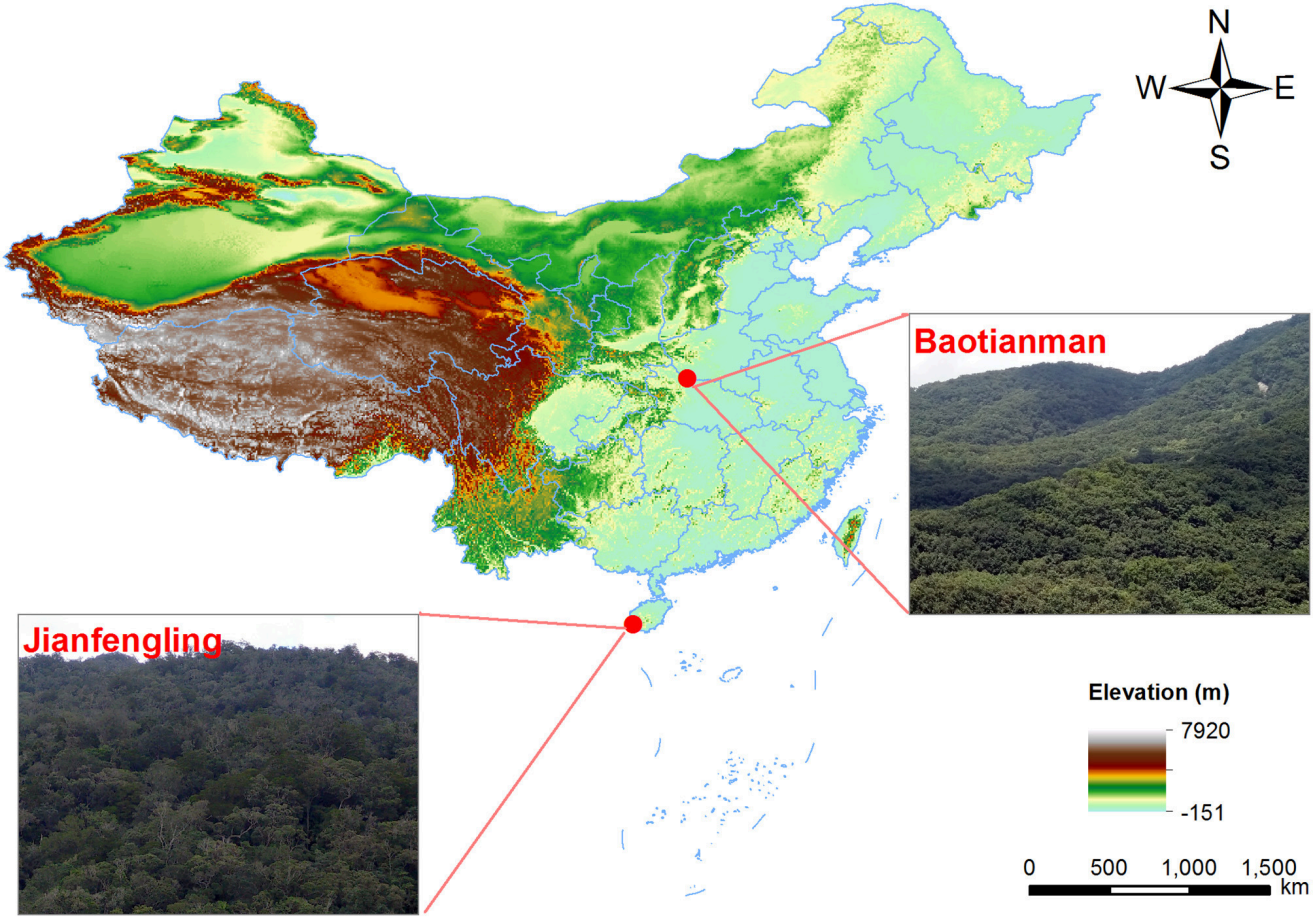
Map of the study areas. Baotianman National Natural Reserve in Neixiang County, Henan Province, and Jianfengling National Natural Reserve in Ledong County, Hainan Province, China.

We chose a temperate deciduous broadleaved forest (BTM) and a tropical mountain rainforest (JFL) as our study sites. BTM is at the east of Qinling Mountains and southern slope of Funiu Mountains, located in the southwest of Henan Province of China (33°20′-33°36′N, 111°47′-112°04′E) (Figure 1). It is in a transitional region which from a northern subtropical climate to a warm temperate climate (Zhao et al., 2014) and is considered to be more sensitive to disturbances and susceptible to climate changes, especially global warming (di Castri and Hansen, 1992). There is a continental monsoon climate with four distinctive seasons at BTM. *Quercus aliena* var. *acuteserrata*, Q. *glandulifera* var. *brevipetiolata* Nakai, *Q*. *variabilis, Carpinus cordata, Cornus controversa*, and *Tilia americana* are the dominant tree species (Luan et al., 2011, 2014; Liu Y. C. et al., 2013). The mean annual precipitation and air temperature is 885.6 mm and 15.1°C, respectively (Liu Y. C. et al., 2013). JFL is located in the southwest of Hainan Province of China (18°23′-18°52′N, 108°36′-109°05′E) (Figure 1), at the northern edge of tropical Asia and has the best-protected tropical mountain rainforests (Yang Q. et al., 2017). It is a tropical mountain rainforest located at a transitional region between subtropical evergreen broad-leaved forest and tropical rainforest. There are distinct rainy and dry seasons at JFL. The mean of annual air temperature is 19.8°C and the mean annual rainfall is 2,449 mm. *Gironniera subaequalis, Cryptocarya chinensis, Livistona saribus*, and *Mallotus hookerianus* are the dominant trees (Chen et al., 2010; Bai et al., 2014).

### Soil sampling

From September of 2015 to June of 2016, the soil samples were collected four times during our study period. September and December of 2015, March and June of 2016 represent autumn, winter, spring, and summer, respectively. We collected soil samples for each season. A total of 20 soil samples at each site (5 replicates per season × 4 seasons) were collected from each site at 0–10 cm below the soil surface after the litter layer was removed. Each replicate was from a composite of three soil samples and was divided into three subsamples. Each of the subsamples was used for different analyses. Soil samples were packed in sterile plastic bags and preserved in an ice-cooled box, which was then transported to the laboratory for further analysis. The soil samples were pre-treated after removal of the surface soil animal and plant residues and sieved through a 2-mm screen. One subsample was placed at −80°C for molecular analysis. One subsample air dried and placed at room temperature to measure total carbon, total nitrogen, pH, and water-filled pore space (WFPS). One subsample was stored in a 4°C refrigerator for measurement of microbial biomass carbon (MBC), microbial biomass nitrogen (MBN), ammonium-nitrogen (${\text{NH}}_{4}^{+}$-N), nitrate-nitrogen (${\text{NO}}_{3}^{-}$-N), and dissolved organic carbon (DOC).

Soil gravimetric moisture content was measured with the oven-drying method at 105°C for 48 h to constant weight. Soil temperature (T) below the top soil at 10 cm was measured by a geothermometer when collecting soil samples. Total nitrogen (TN) was detected by the Kjeldahl method using a Kjeltec 8400 Analyzer (FOSS, Sweden). The dissolved organic carbon (DOC), ammonium nitrogen (${\text{NH}}_{4}^{+}$-N) and nitrate nitrogen (${\text{NO}}_{3}^{-}$-N) were measured as previously described extracted with 2 mol/L KCl and their concentrations were analyzed using a continuous-flow analyzer (San++, Skalar, the Netherlands). The pH was measured through a Sartorius pH meter (PB-10, Germany) with deionized water (soil: water ratio of 1:2.5). Total carbon (TC) contents and microbial biomass carbon (MBC) in soil samples were estimated through the chloroform fumigation-extraction method (Joergensen, 1996) on a TOC analyzer (LIQUIC TOCII, Elementar Analyse systeme GmbH, Hanau, Germany). Volumetric soil water content was measured and changed to soil water-filled pore space (WFPS) via soil porosity data.

### Sequencing

We used CTAB/SDS method to extract total DNA from soil samples. Using the 1% agarose gels to detect DNA concentration and then the DNA concentration was diluted to 1 ng/μL. The genes of 16S V3-V4 distinct regions were amplified used specific primer 515F-806R with the barcode (Gungor et al., 2016). All PCR reactions were operated with Phusion®High-Fidelity PCR Master Mix (New England Biolabs). Added buffer (1X loading including SYB dye) with PCR products and carried out electrophoresis using 2% agarose gel. The 400–450 bp PCR products were selected for analysis of population structure. We mixed up the PCR products in equal density ratios and then purified with an Extraction Kit (Qiagen, Germany) and generated sequencing libraries via DNA PCR-Free Sample Preparation Kit (Illumina TruSeq®, USA) according to the manufacturer's instructions. To evaluate library quality, the Thermo Scientific Qubit@ 2.0Fluorometerand Agilent Bioanalyzer 2100 system were used. Then 250 bp paired-end reads was produced via the Illumina HiSeq 2500.

### Data analysis

Based on unique barcode, the paired-end reads was operated and merged using the FLASH software (Magoc and Salzberg, 2011). We adjusted the maximum mis-match density to 0.1 in FLASH and the other parameters used their default values. Under specific filtering conditions, the quality filtering on the raw tags were performed to get the high-quality clean tags according to the QIIME quality controlled process (Caporaso et al., 2010). The tags were compared with the Gold reference database using UCHIM algorithm to remove chimera sequences and obtain the effective tags (Edgar et al., 2011). We accessed the databases on Sept 29, 2016. Sequences analyses were performed by Uparse software (Edgar, 2013). Based on the review of literature (e.g., Yang H. et al., 2017; Zhang and Wang, 2017), we thought that OTUs clustered at 99% may be too conservative. We considered a threshold of 97% to be practical from the perspective of accessing bacterial diversity and composition in forest soils. Sequences with ≥97% similarity were assigned to the same OTUs. Typical sequence for every OTU was screened for further annotation. For each representative sequence, the Greengenes Database was used for RDP classifier. The threshold classification score is about 0.8–1 used for taxonomic assignment with the RDP classifier. The abundance information of OTUs was normalized using the smallest numbers of sequences of 31,708 (with singletons removed) (Wen et al., 2017). The Shannon index was used to estimate species diversity of the soil bacterial community across two forests with QIIME (Version1.7.0) and displayed with R software (Sun et al., 2014). To understand which soil physiochemical properties influence the bacterial communities in the two forest soils, we performed multivariate redundancy analysis (RDA). We used the Type I scaling RDA to analyzed the correlative relationships between variables (OTUs and physiochemical properties of soil) (Buttigieg and Ramette, 2014).

Differences in function group abundance between two forests were compared with the software FAPROTAX (Louca et al., 2016). ANOVA analyses of differences were performed using SPSS software in taxonomic order abundance. All statistical analyses were assessed at α = 0.05.

Sequence data was deposited at the National Center for Biotechnology Information (NCBI) Sequence Read Archive database (accession number: SRP130793).

## Results

### Structure of soil bacterial community

After multiple levels of quality control to filter the raw reads, we obtained 59,385 paired-end reads from the 40 soil samples. After joining, removing the low quality of sequence, the remaining 55,871 tags sequence was used for the subsequent analyses. A total of 31,708 OTU clustering at 97% sequence identity was mapped to 556 known genera. For the BTM, the order of seasons in which the most abundant OTUs were detected was spring >winter > summer >autumn. For the JFL, the order was summer > winter > autumn > spring.

**Figure 2:**
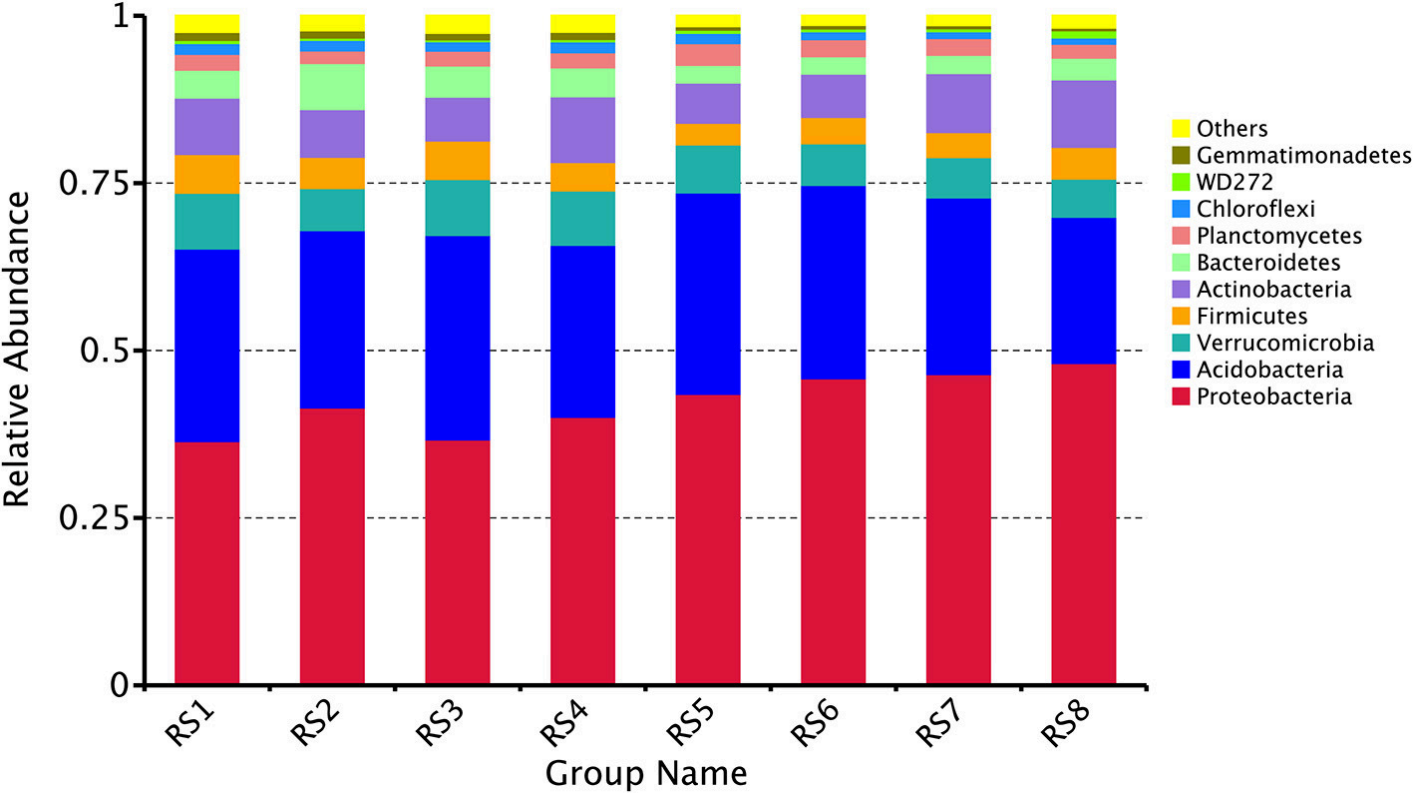
Community composition in two forests soil at the phylum level. RS1-2: autumn and winter of 2015 at Baotianman; RS3-4: spring and summer of 2016 at Baotianman; RS5-6: autumn and winter of 2015 at Jiangfengling; RS7-8: spring and summer of 2016 at Jiangfengling.

In the two forests sites, bacterial OTUs were assigned to 44 different phyla. Ten different phyla (*Proteobacteria, Acidobacteria, Verrucomicrobia, Firmicutes, Actinobacteria, Bacteroidetes, Planctomycetes, Chloroflexi, WD272, Gemmatimonadetes*) together comprised more than 90% of the relative abundance in each library. The bacterial communities demonstrated marked differences in taxon richness and relative abundance patterns at both phylum taxonomic classification levels. At BTM, *Proteobacteria* were the most abundant at phylum level (mean relative abundance of 38.7%), followed by *Acidobacteria* (27.9%), *Verrucomicrobia* (7.8%), *Firmicutes* (5.1%), *Actinobacteria* (8.0%), *Bacteroidetes* (5.0%), and *Planctomycetes* (2.2%). While at JFL, *Proteobacteria* were the most abundant phylum (46%), followed by *Acidobacteria* (26.8%), *Verrucomicrobia* (6.3%), *Firmicutes* (3.9%), *Actinobacteria* (7.8%), *Bacteroidetes* (2.8%), and *Planctomycetes* (2.6%) (Figure 2). At the class level, the abundance of top 10 classes had no significant difference throughout the year in each site and there was no significant difference between BTM and JFL in the same season either (Supplementary Data Sheet S1).

### Relationship between bacterial community structure and environmental variables

**Figure 3:**
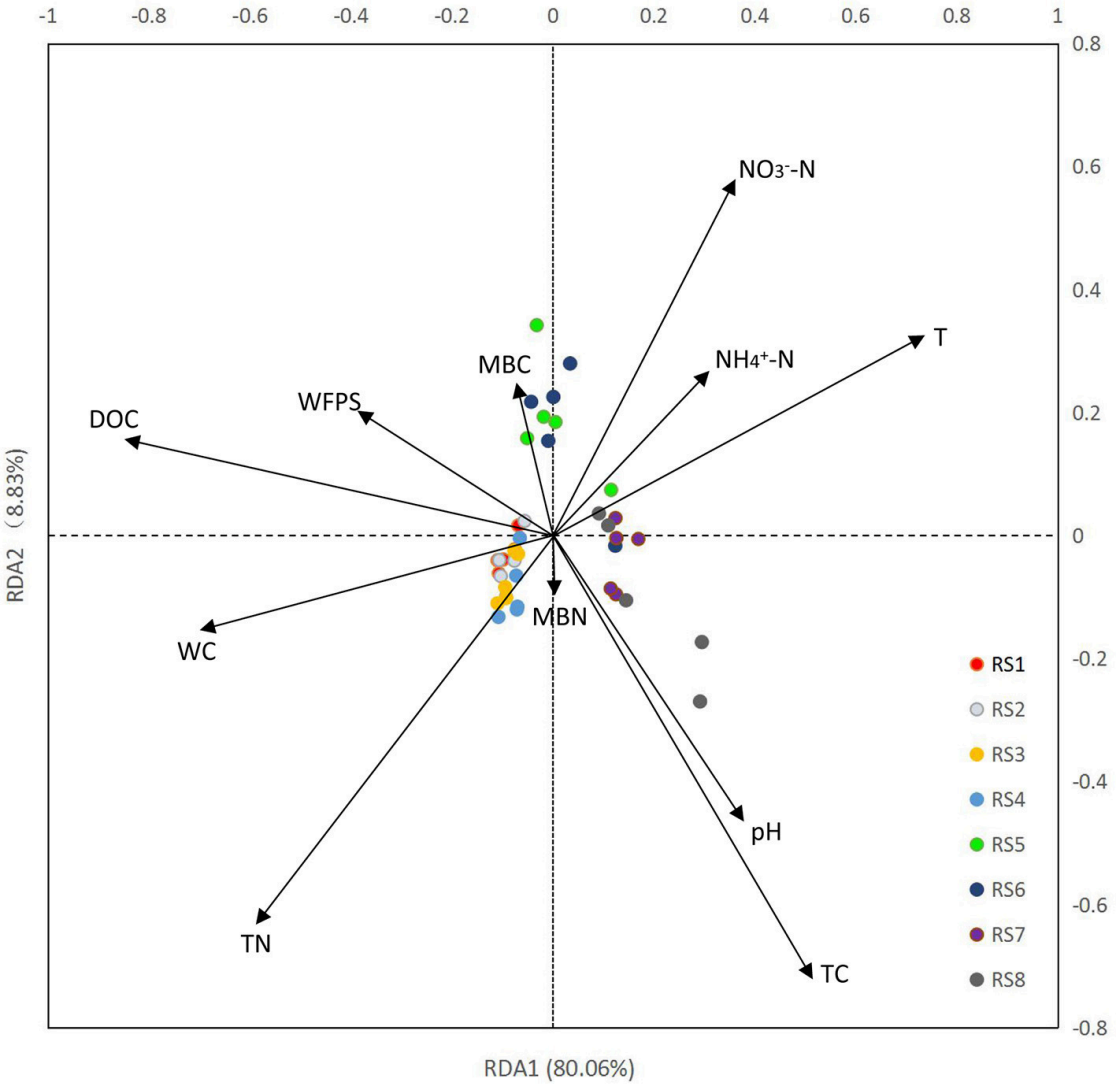
Redundancy analysis (RDA) of high-throughput sequencing data with soil physiochemical properties. WC, water content; T, temperature; WFPS, water-filled pore space; ${\text{NO}}_{3}^{-}$-N, nitrate nitrogen; ${\text{NH}}_{4}^{+}$-N, ammonia nitrogen; DOC, dissolved organic carbon; TC, total carbon; TN, total nitrogen; MBN, microbial biomass nitrogen; and MBC, microbial biomass carbon. Arrow lengths indicate the strength of the relationship between the soil property and the overall microbial community. The direction of the line indicates the direction of increase for a specific soil physiochemical property.

Results of RDA showed a clear association between bacterial community structure and soil physiochemical properties (Figure 3). In total, 11 physicochemical properties, including soil water content (WC), T, WFPS, pH, TC, TN, DOC, ${\text{NH}}_{4}^{+}$-N, ${\text{NO}}_{3}^{-}$-N, MBN, and MBC were measured in our study (Table 1). The first ordination RDA axis (RDA1) was mainly correlated with DOC, T, WC, and explained 80.06% of the total variability of the bacterial community structure. The second ordination axis (RDA2), which was strongly associated with TC, TN, ${\text{NO}}_{3}^{-}$-N, explained 8.83% of the total variability. RDA1 was positively correlated with T, pH, ${\text{NO}}_{3}^{-}$-N, TC, ${\text{NH}}_{4}^{+}$-N and negatively correlated with WC, WFPS, DOC, TN, MBC. As important variables (represented by longer arrows), DOC, T, WC played vital roles in the shaping of bacterial community at our two study sites.

**Table 1:** Soil physicochemical properties at BTM and JFL during different seasons.

**Soil property**	**Site**	**WC (%)**	**T (°C)**	**pH**	**WFPS (%)**	**TC (g/kg)**	**TN (g/kg)**	**${\text{NH}}_{4}^{+}$-N (mg/kg)**	**${\text{NO}}_{3}^{-}$-N (mg/kg)**	**MBC (g/kg)**	**DOC (g/kg)**	**MBN (mg/kg)**
Autumn(2015)	BTM	33.7 ± 2.5ab	12.6 ± 0.6b	4.7 ± 0.1b	37.2 ± 3.6b	32.3 ± 2.9c	2.29 ± 0.15c	13.5 ± 0.7a	2.6 ± 0.2a	0.54 ± 0.08a	1.62 ± 0.17a	43.5 ± 6.8b
	JFL	26.4 ± 1.6a	22.5 ± 0.1b	4.9 ± 0.0b	52.5 ± 1.7a	40.0 ± 1.6c	2.45 ± 0.15a	20.0 ± 4.7a	5.6 ± 1.0a	0.40 ± 0.04b	1.50 ± 0.31a	126.4 ± 40.3b
Winter(2015)	BTM	41.5 ± 2.8a	−0.6 ± 2.2*d*	4.1 ± 0.2c	51.9 ± 3.8ab	77.4 ± 8.0a	3.84 ± 0.33b	11.3 ± 2.9ab	2.1 ± 0.2ab	0.54 ± 0.15a	1.84 ± 0.14a	171.0 ± 52.4a
	JFL	22.4 ± 0.6b	17.7 ± 0.2c	4.2 ± 0.1c	43.6 ± 3.0b	43.6 ± 3.5c	2.29 ± 0.07a	8.9 ± 0.5a	5.9 ± 1.2a	0.71 ± 0.08a	1.36 ± 0.05a	85.4 ± 21.3b
Spring(2016)	BTM	28.6 ± 1.5b	6.4 ± 1.2c	5.1 ± 0.1a	50.3 ± 4.3b	61.3 ± 2.5b	4.21 ± 0.22ab	8.1 ± 0.4b	1.6 ± 0.2b	0.35 ± 0.08a	1.61 ± 0.12a	190.7 ± 36.95a
	JFL	22.2 ± 2.0ab	17.2 ± 0.3c	5.1 ± 0.1ab	34.1 ± 1.2c	62.2 ± 3.2b	2.24 ± 0.03a	12.1 ± 5.2a	1.1 ± 0.3b	0.51 ± 0.05ab	0.34 ± 0.03b	219.4 ± 16.8a
Summer(2016)	BTM	21.9 ± 1.9c	17.0 ± 0.5a	5.2 ± 0.1a	60.4 ± 2.9a	62.0 ± 1.6b	4.74 ± 0.30a	7.3 ± 0.8b	1.0 ± 0.1b	0.36 ± 0.09a	1.62 ± 0.05a	83.8 ± 35.6ab
	JFL	21.2 ± 2.9b	24.7 ± 0.1a	5.3 ± 0.1a	41.9 ± 1.9b	121.5 ± 5.7a	2.34 ± 0.06a	14.1 ± 3.6a	5.7 ± 0.8a	0.31 ± 0.09b	0.53 ± 0.04b	89.4 ± 13.5b

*Different letters indicate significant difference between different seasons at the same site (P < 0.05)*.

### The shannon diversity of soil bacterial community

**Figure 4:**
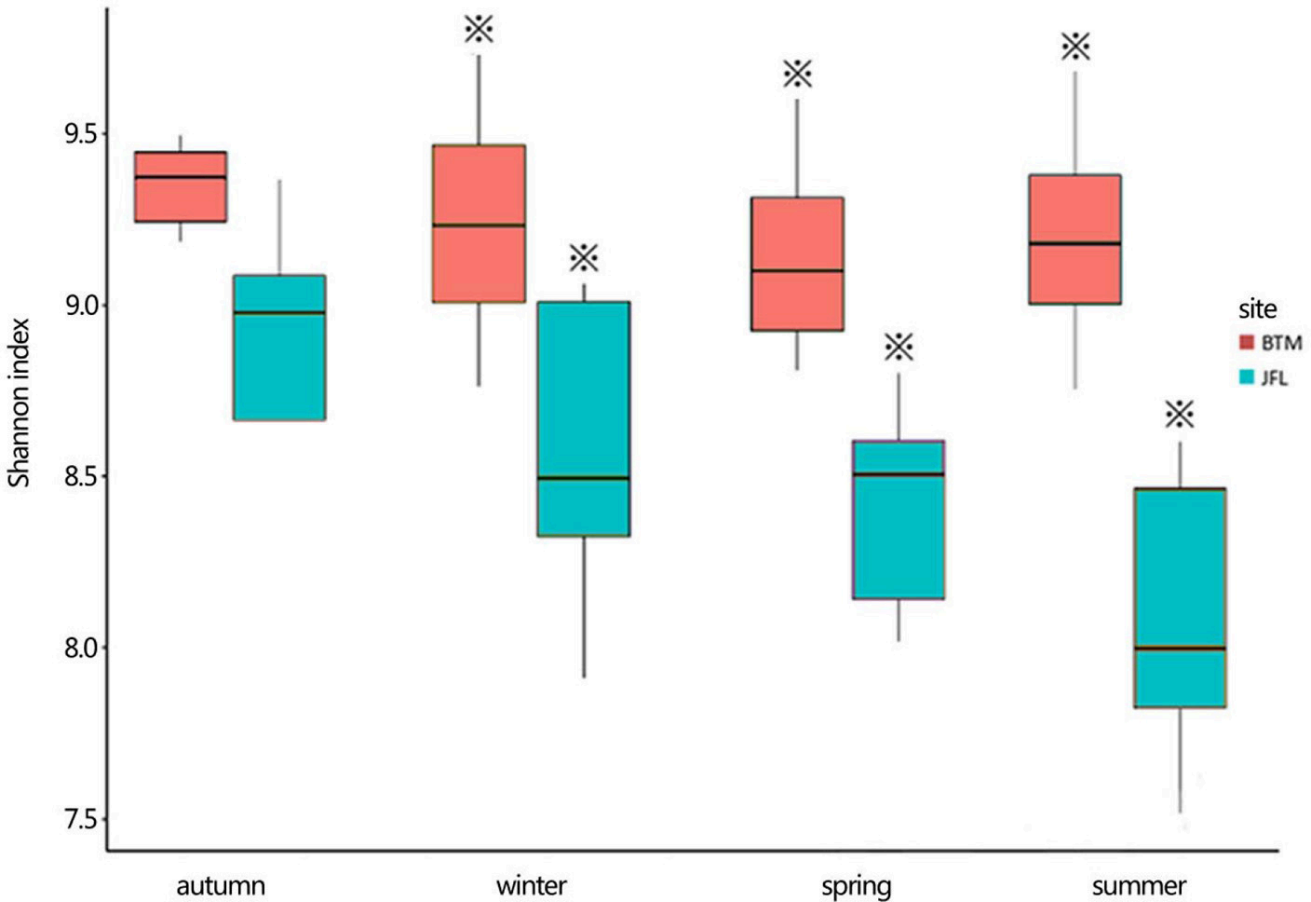
The bacterial Shannon index of the two forest sites. Autumn and winter represent the autumn and winter of 2015, respectively. Spring and summer represent the spring and summer of 2016, respectively. The asterisks represent significant differences between BTM and JFL at the same season (*P* < 0.05).

The Shannon diversity index at BTM was ~9.2, the highest and lowest bacterial Shannon index at all 20 sampling was 9.755 and 9.305, respectively (Figure 4). Whereas, the Shannon diversity index at JFL was between ~8.0 and 9.0, the highest and lowest bacterial Shannon index was 8.79 and 7.52, respectively. Overall, the bacterial communities at BTM presented higher diversity values. From September of 2015 to June of 2016, Shannon index did not significantly alter in four seasons at BTM and JFL. Except for September of 2015, the Shannon index had significant differences in the same season between BTM and JFL (*P* < 0.05 in December and *P* < 0.01 in March and June of 2016). At BTM, TN was negatively correlated with the Shannon index (*P* < 0.05). The Shannon index at JFL was related to the WFPS, DOC, TC (*P* < 0.05). WFPS and DOC were positively correlated with the Shannon index, while TC was negatively correlated with the Shannon index (Table 2).

**Table 2:** The relationship between Shannon index and soil physical and chemical properties.

**Site**	**WC**	**T**	**pH**	**WFPS**	**${\text{NO}}_{3}^{-}$-N**	**${\text{NH}}_{4}^{+}$-N**	**DOC**	**TC**	**TN**	**MBN**	**MBC**
BTM	0.297	0.048	0.122	0.252	0.351	0.104	−0.234	−0.045	−0.447*	−0.090	−0.033
JFL	0.396	−0.188	−0.275	0.469*	0.176	−0.195	0.445*	−0.557*	−0.381	0.117	0.060

Asterisks indicate significant correlation between Shannon index and various physical and chemical factors in soils (*P < 0.05). All data from four seasons at the two sites (n = 20). WC, water content; T, temperature; WFPS, water-filled pore space; ${\text{NO}}_{3}^{-}$-N, nitrate nitrogen; ${\text{NH}}_{4}^{+}$-N, ammonia nitrogen; DOC, dissolved organic carbon; TC, total carbon; TN, total nitrogen; MBN, microbial biomass nitrogen and MBC, microbial biomass C.

### Bacterial functions

**Figure 5:**
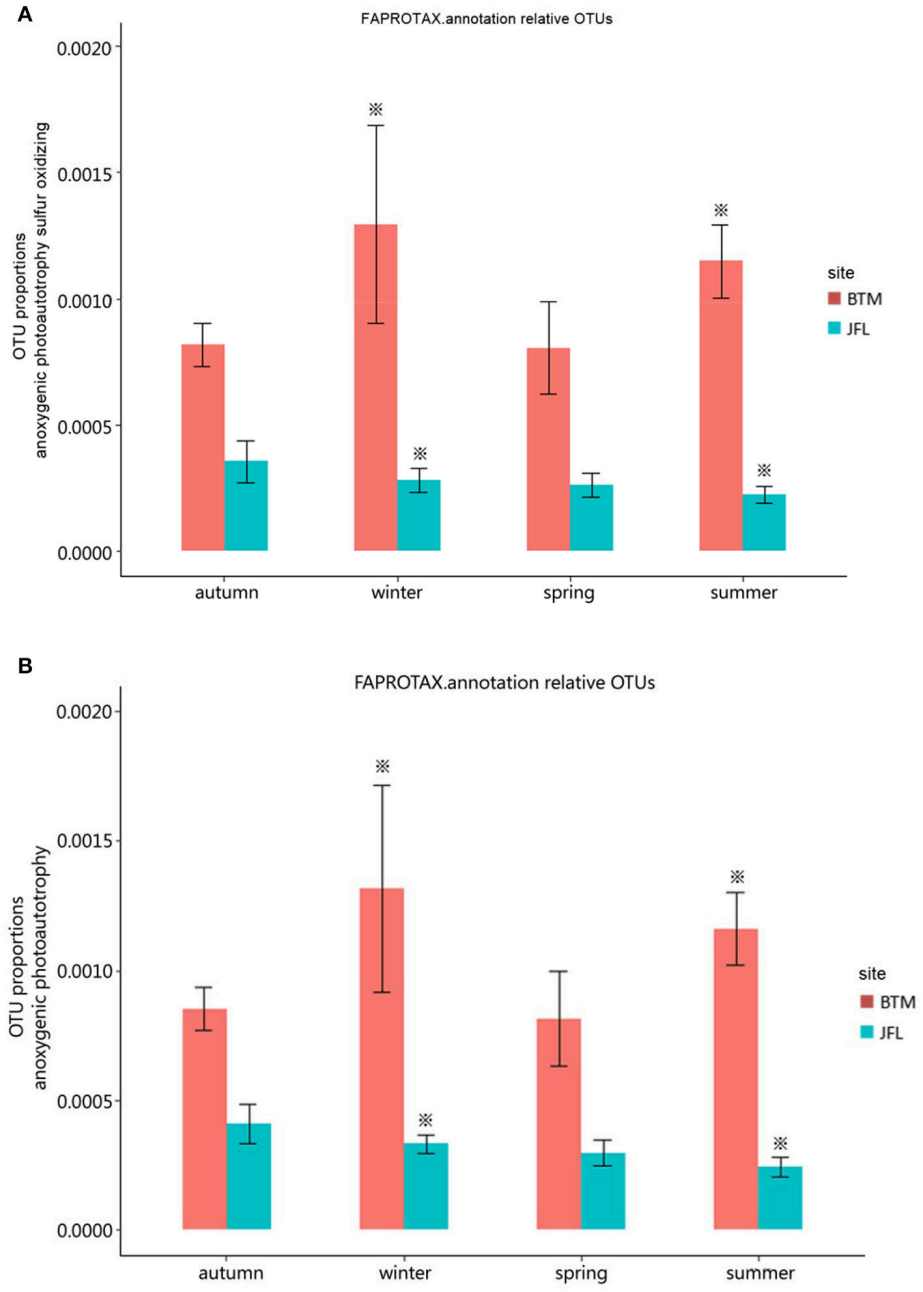
Functional groups with significant difference in carbon cycle. Autumn and winter represent the autumn and winter of 2015, respectively. Spring and summer represent the spring and summer of 2016, respectively. The asterisks represent significant differences between BTM and JFL at the same season (*P* < 0.05). **(A)** OTU proportions of anoxygenic photoautotrophy sulfur oxidizing. **(B)** OTU proportions of anoxygenic photoautotrophy.

We detected eight ecological function groups related to the carbon cycles. They were (1) methanol oxidation, (2) methanotrophy, (3) methylotrophy, (4) phototrophy, (5) photoautotrophy, (6) anoxygenic photoautotrophy H_2_ oxidizing, (7) anoxygenic photoautotrophy sulfur oxidizing, and (8) anoxygenic photoautotrophy (Supplementary Table S1). The anoxygenic photoautotrophy sulfur oxidizing and anoxygenic photoautotrophy pathways were found to show significant differences in the winter of 2015 and the summer of 2016 between BTM and JFL (*P* < 0.05). Others related to carbon cycles were not significant at the same season between two sites (*P* > 0.05) (Figures 5A,B).

**Figure 6:**
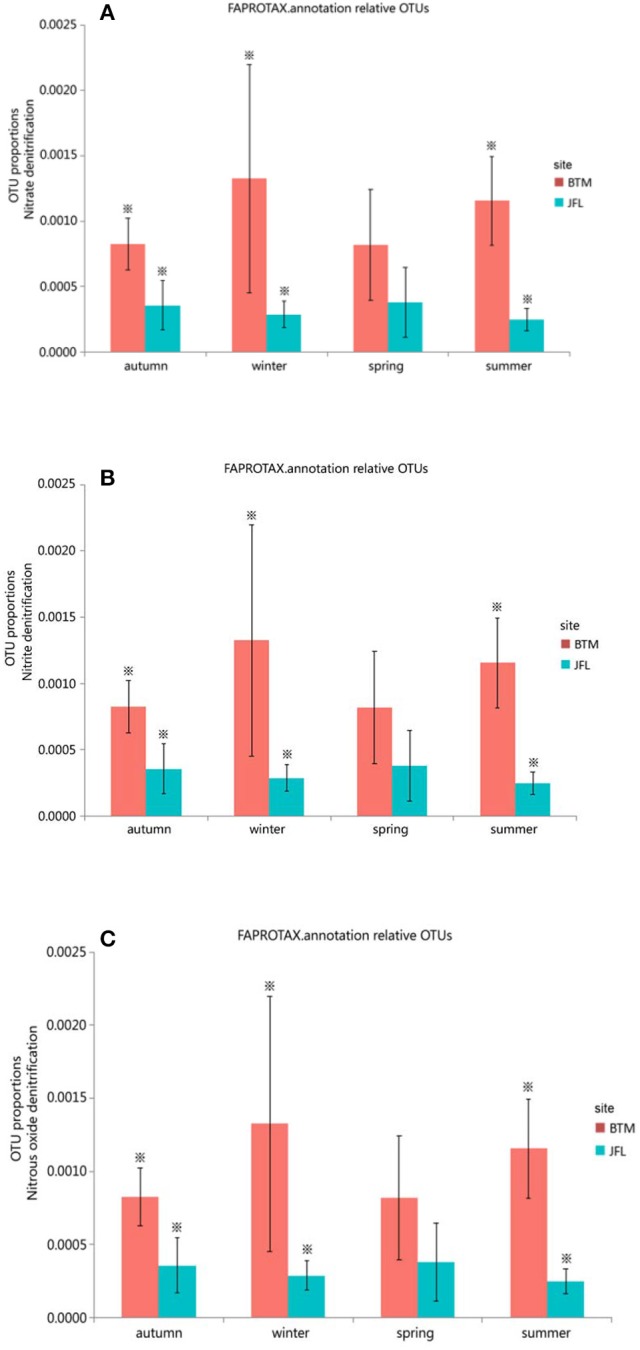
Functional groups with significant difference in nitrogen cycle. Autumn and winter represent the autumn and winter of 2015, respectively. Spring and summer represent the spring and summer of 2016, respectively. The asterisks represent significant differences between BTM and JFL at the same season (*P* < 0.05). **(A)** OTU proportions of nitrous denitrification. **(B)** OTU proportions of nitrite denitrification. **(C)** OTU proportions of nitrous oxide denitrification.

We also detected 12 ecological function groups related to the nitrogen cycles. They were (1) aerobic ammonia oxidation, (2) aerobic nitrite oxidation, (3) nitrate denitrification, (4) nitrate reduction, (5) nitrate respiration, (6) nitrification, (7) nitrite ammonification, (8) nitrite denitrification, (9) nitrite respiration, (10) nitrogen fixation, (11) nitrogen respiration, and (12) nitrous oxide denitrification (Supplementary Table S1). However, only three groups (nitrate denitrification, nitrite denitrification, and nitrous oxide denitrification) showed significant differences in the autumn and winter of 2015 and the summer of 2016 between BTM and JFL (*P* < 0.05). Other functions were not significantly different at the same season between two sites (*P* > 0.05) (Figures 6A–C).

There was no significant difference in terms of carbon and nitrogen cycles among the four seasons at BTM or JFL (*P* > 0.05) (Figures 5A,B, 6A–C).

**Figure 7:**
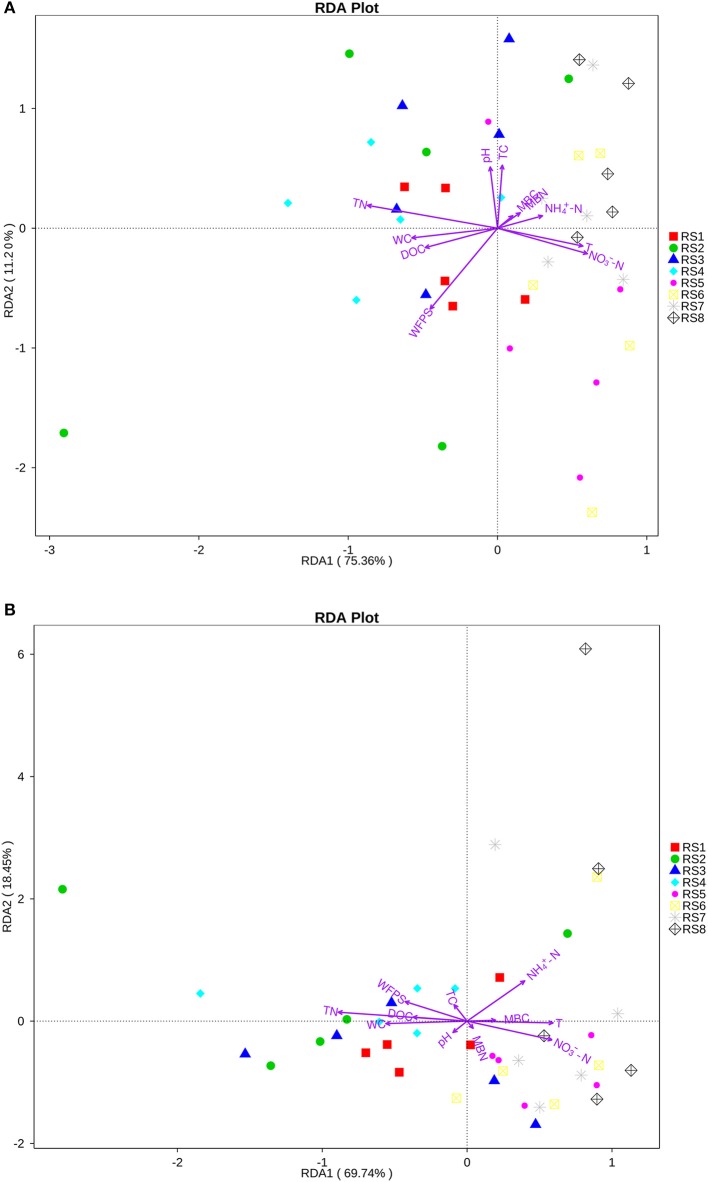
Redundancy analysis (RDA) of the predicted FAPROTAX functions asscoated with soil physiochemical properties.**(A)** Functional groups that are related to carbon cycle. **(B)** Functional groups that are related to nitrogen cycle. WC, water content; T, temperature; WFPS, water-filled pore space; ${\text{NO}}_{3}^{-}$-N, nitrate nitrogen; ${\text{NH}}_{4}^{+}$-N, ammonia nitrogen; DOC, dissolved organic carbon; TC, total carbon; TN, total nitrogen; MBN, microbial biomass nitrogen; and MBC, microbial biomass carbon. Arrow lengths indicate the strength of the relationship between the soil property and the overall microbial community. The direction of the line indicates the direction of increase for a specific soil physiochemical property. RS1-2: autumnand winter of 2015 at Baotianman; RS3-4: springandsummerof 2016 at Baotianman; RS5-6: autumn and winter of 2015 at Jiangfengling; RS7-8: spring and summer of 2016 at Jiangfengling.

Figure 7A is the RDA analysis of environmental factors and functional groups related to the carbon cycle, Figure 7B is the RDA analysis of environmental factors and functional groups related to the nitrogen cycle. The Figure 7A showed that the first axis (RDA1) explained 75.36% of the variation in terms of the relative abundances of functional groups variability related to the carbon cycle. TN, NO_3_-−N, and WC are the dominated environmental factors in the first axis, which in total explained 48.8% of the changes in functions related to carbon cycle. In addition, the Figure 7B showed that the first axis (RDA1) explained 69.74% of the variation in the relative abundances of functional groups variability related to the nitrogen cycle. TN, T, ${\text{NO}}_{3}^{-}$-N were the dominated environmental factors in the first axis, which in total explained 48.6% of the changes in functions related to nitrogen cycle (Figures 7A,B).

In Figure 7A,B, DOC mainly contributed to the first axis, where the two samples sites were separated. DOC contributed 11.6% to the first axis in Figure 7A and 8.8% in Figure 7B. DOC was closely related to soil temperature and water content.

## Discussion

### Bacterial community comparison and diversity

Our results demonstrate that *Proteobacteria* (accounting for 38.7 ± 1.2% at BTM and 46.0 ± 1.0% at JFL) and *Acidobacteria* (accounting for 27.9 ± 1.1% at BTM and 26.8 ± 1.8% at JFL, respectively) were the dominant bacterial phyla in the two forest soils. In terms of bacterial classification, the bacterial community did not differ greatly between the JFL tropics forest and BTM temperate forest, including relatively high abundances of *Proteobacteria* and *Acidobacteria*. With *Proteobacteria* being the most abundant phylum in the two forest soils, our result is consistent with previous studies (Bastian et al., 2009; Li et al., 2014). *Actinobacteria, Planctomycetes*, and *Chloroflexi* were also abundant phyla. But our findings are inconsistent with the results of Liu who found that *Actinobacteria* play a dominant role in the bacterial community of the mixed forest soil in Dinghushan Mountain (Liu L. et al., 2013). Janssen reviewed that *Proteobacteria* and *Acidobacteria* dominated the bacterial community of different geographic regions and soil types (Janssen, 2006). Our results are in agreement with Janssen. Both *Proteobacteria* and *Acidobacteria* are the major bacterial community, although BTM and JFL are located in different climatic regions with different environmental conditions. Our results are similar to the result of previous studies (Miyashita, 2015). It is found that members of *Proteobacteria* and *Acidobacteria* are ubiquitous to almost all soil types (Zhang and Xu, 2008).

Despite large differences in aboveground conditions at BTM (a temperate forest) and JFL (a tropical rainforest), the composition of bacterial groups were similar across two sites for all phyla detected. Bacterial diversity was unrelated to site temperature, latitude, and other variables. It may indicate that the community composition was largely independent of geographic distance (Fierer and Jackson, 2006).

### Effects of soil physiochemical characteristics on bacterial community composition and diversity

Temperature affected the bacterial compositions in previous studies (Wells et al., 2009; Wang et al., 2012; Ligi et al., 2014). Temperatures are the primary seasonal characteristics in winter and summer at BTM. The environments have long been considered to force microorganisms into a dormant state in winter (Uchida et al., 2005). Bacteria can survive and keep active at subzero or extremely cold temperatures (Capenter et al., 2000; Junge et al., 2002). Some groups of bacteria are important members of the overall microbial communities and active participant in the process of foliar litter composition (Bergfur and Sundberg, 2014). In general, low temperatures lead to less microbial diversity and even kill some bacteria species who have low cold-tolerance (Zhao et al., 2016). In winter, some bacteria might been dead and the bacterial Shannon index was reduced, but certain bacterial species might improve their superiority for their high tolerance of cold conditions in the community (Walker et al., 2006; Zhao et al., 2016). Studies have documented that soil temperature could change the microbial community composition (Zhang et al., 2005). In our study period, there was no relationship between temperature and Shannon index in four seasons at BTM and JFL (Table 2). Some studies have shown temperature can affect the alpha diversity of bacterial community structure quickly. However, significant changes may not be always observed in the short term (Stark et al., 2007).

Soil moisture content differed significantly between two sites. The high moisture condition at BTM had impacted bacteria communities, causing the higher diversity. Ahn and Peralta found that consistently higher diversity was in the dry site (Ahn and Peralta, 2009). In our study, water content had no relationship with Shannon index at BTM and JFL. In many habitats, previous studies also confirmed that water content is an important factor in controlling the community structure and diversity of bacteria (Lauber et al., 2009; Nemergut et al., 2010; Brockett et al., 2012). The water content can impact the availability of water for the bacteria as well which can affect the enzyme activities.

In our study, DOC affected bacterial community structure (Figure 3). DOC is an actively carbon pool driving the decomposition processes and is the direct precursor of bacterial growth and activity (Roesch et al., 2007). The main environmental processes can be affected by the effectiveness of DOC, such as facilitating the transport of N, P, S, and energy supply to bacteria (Roesch et al., 2007). At JFL, the favorable temperature and humidity and adequate nutrient a whole year supply that provide good conditions for most decomposers in the decomposition of litter. Bacterial diversity clearly declined along with increasing nitrogen content (Campbell et al., 2010). Due to the loss of plant species diversity, the bacterial diversity declined (Phoenix et al., 2012). The forest sites are another potential DOC pool that might influence the bacterial community compositions. Bacteria are more limited by water than fungi (Guhr et al., 2015). For the better adaptation, bacteria can lower soil water potentials in their strong cell walls and prevent water losses (Guhr et al., 2015).

In this study, the soil was slightly acidic (pH of 4–5) in two study sites. Our two forest sites without remarkable human disturbance (Such as fertilizing, warming, etc.), so the pH and bacterial communities all had no significant change in the four seasons. Soil pH was considered to be one of the main factors forming bacterial community structure (Preem et al., 2012; Bergkemper et al., 2016) and affectingmicrobial activity (Truu et al., 2008; Bergkemper et al., 2016). Several studies have shown that the proportion of bacteria from *Acidobacteria* were sensitive to pH (Jones et al., 2009; Chu et al., 2010). The bacterial communities generally have neutral pH in the arid and semiarid soils, as bacterial communities from temperate and tropical forest soils with generally acidic (Fierer and Jackson, 2006). Our results showed that the soil pH had no relationship with Shannon index (Table 2).

Bacterial community composition and diversity, which were closely related with soil and vegetation types (Cong et al., 2015). Maybe, the structure and diversity of bacterial composition are determined by the cooperation of various physical and chemical factors, vegetation properties at BTM and JFL (Figure 4).

### Functional analysis of the bacterial community

Soil bacterial communities provide important biogeochemical cycles (Jenkins et al., 2017). While members of *Chloroflexi* take part in photoautotrophic carbon fixation (Klatt et al., 2013), the heterotrophic *Bacteroidetes*, and *Firmicutes* are related to the decomposition of soil organic matter (Bolhuis et al., 2014). The decomposition of specific carbon substrates could be influenced by community composition of soil bacteria with different resource requirement, those differences may lead to the differences in dissolved organic matter decomposition rates at the two forest sites (Leff et al., 2015). Along with the seasonal allocation of photosynthesis products to plant roots, the effectiveness of carbon also changes seasonally in topsoil (Žifčáková et al., 2017). There was significant difference in anoxygenic photoautotrophy sulfur oxidizing and anoxygenic photoautotrophy at BTM and JFL in winter of 2015 and in summer of 2016 (*P* < 0.05). This may be due to the differences in temperature and water between BTM and JFL in winter and summer, respectively.

The soil nitrogen cycle is related with greenhouse gases, such as the production and emission of CO_2_, CH_4_, and N_2_O (Johnson et al., 2005). Meanwhile, the seasonal differences in carbon and nitrogen utilization and bacteria associated with the process of carbon and nitrogen cycles (Žifčáková et al., 2017). Bacteria play an important role in soil nitrogen cycle, such as ammonification, nitrogen fixation, denitrification, and nitrification (Yoon et al., 2015). There was significant difference in nitrate denitrification, nitrite denitrification and nitrous oxide denitrification at BTM and JFL (*P* < 0.05). This may be due to the effect of multiple environmental factors.

From Table 1, we can see the DOC showed significant differences at the spring and summer of 2016 between BTM and JFL, but not in the autumn and winter of 2015 between the two sites. Optimal moisture and temperature conditions results in an enhanced production of DOC in the forest soils (Michalzik et al., 2001). The determination of factors impacting the forest soil DOC is vital for the prediction of organic carbon and nitrogen pools in soils (Michalzik et al., 2001). In forest soils, the seasonality of DOC concentrations was related to carbon and nitrogen input from above-ground litter that affects the function groups of the nitrogen and carbon cycles (Currie et al., 1996; Gundersen et al., 1998).

Anoxygenic photosynthesis is performed by various purple and green bacteria with bacteriochlorophylls. They cannot produce oxygen. Depending on the different carbon sources, anoxygenic phototrophic microorganisms are classified into photoautotrophs and photoheterotrophs. The proteobacteria is the largest group of photosynthetic bacteria (Stackebrandt et al., 1996). The shift of oxic conditions and water content may increase the distribution of anoxygenic photoautotrophic production (Dong et al., 2015).

In the extent, bacterial denitrifier community is crucial to nitrogen cycle in soil, meanwhile the relative abundance of specific OTUs are more informative when predicting the community functions (Bent et al., 2016). Nitrate denitrification, nitrite denitrification, and nitrous oxide denitrification related to nitrogen cycle showed a significant difference between BTM and JFL at the same season (*P* < 0.05). The reason for this difference may be due to the presence of the denitrification genes (nirS, nirK, and nosZ) in soil microorganisms, which was significantly different at the same season for two forest sites and varied with DOC or water content.

There was no significant difference (*P* > 0.05) across four seasons at BTM and JFL for methanol oxidation, methanotrophy, methylotrophy, phototrophy, photoautotrophy, anoxygenic photoautotrophy H_2_ oxidizing, aerobic ammonia oxidation, aerobic nitrite oxidation, nitrate reduction, nitrate respiration, nitrification, nitrite ammonification, nitrite respiration, nitrogen fixation, and nitrogen respiration. The bacterial community associated with these functions were not significantly different between the two sites. Anoxygenic phototrophic bacteria are considered to utilize simple inorganic compounds, simple organic acids and alcohols for photoautotrophic growth (Tran, 2008). It seems that there were significant differences in the amount of these compounds at the same season between the two sites, leading to some differences in these functions.

To our knowledge, this is the first attempt to detect bacterial community composition and function in soil at BTM and JFL. The results showed that there was clear soil bacterial functional redundancy at BTM and JFL. These results may be explained in two ways. The first is that the soils at the forest sites may have similar functions for the carbon, nitrogen cycles. The second is that bacteria with close evolutional relationship may have very different environmental tolerances and thus functions. Soil bacteria control soil processes, such as the associated release of greenhouse gases such as CH_4_, N_2_O, and CO_2_ into the atmosphere. The soil of forest contains a lot of microorganisms, including bacteria, archaea, fungi, and so on. This study only examined the bacterial abundance, diversity, and potential function in the soil of BTM and JFL. Genes related to carbon and nitrogen cycles, such as nitrification, carbon and nitrogen fixation, carbon degradation, methane oxidation, and nitrite reduction have been regarded as the most important and should be quantified at both forest sites in our further research. However, the large standard errors for the denitrification plots indicates the limitation in interpreting the statistical significance of abundance differences for those data and this should be better addressed in further study.

## Conclusions

To determine soil bacterial community structure, diversity, and function in different typical forest types, we chosen two natural and mature forest sites in China (the BTM deciduous broad-leaved forest and JFL tropical mountain rainforest). This study compared bacterial community diversity and function in four seasons via high-throughput sequencing. Our results showed that soil bacterial composition at phylum level (top 10 phyla) were similar, excepting for the difference in the relative abundance. BTM and JFL contain a core and stable bacterial community during our study period. Between BTM and JFL, there was significant difference in Shannon index. Here, changing of TN at BTM and changing of WFPS, DOC, TC at JFL appear to correlate with the Shannon index, respectively. The bacteria at two sites showed two significant predicted functions related to carbon cycle and three significant predicted functions related to nitrogen cycle. It is one of the few studies that examine the bacterial diversity, relationship between bacterial community structure and function in two typical forest sites at the climatic transitional regions under natural conditions in China.

## Author contributions

HW, CP, and MW designed the experiment and supervised all work. BY was involved in the experimental design and participated in the field and lab work. HW, DC, YL and XL were involved in the field work. All authors contributed to the preparation and revision of the manuscript.

### Conflict of interest statement

The authors declare that the research was conducted in the absence of any commercial or financial relationships that could be construed as a potential conflict of interest.
