# Supplementary material for: Contrasting Soil Bacterial Community, Diversity, and Function in Two Forests in China
Source: Front Microbiol. 2018 Jul 31;9:1693. doi: 10.3389/fmicb.2018.01693 (PMC6080587; doi:10.3389/fmicb.2018.01693)

Supplementary 1

Community compostion in two forest soils at the class level. RS1-2: autumn and winter of 2015 at Baotianman; RS3-4: spring and summer of 2016 at Baotianman; RS5-6: autumn and winter of 2015 at Jianfengling; RS7-8: spring and summer of 2016 at Jianfengling.

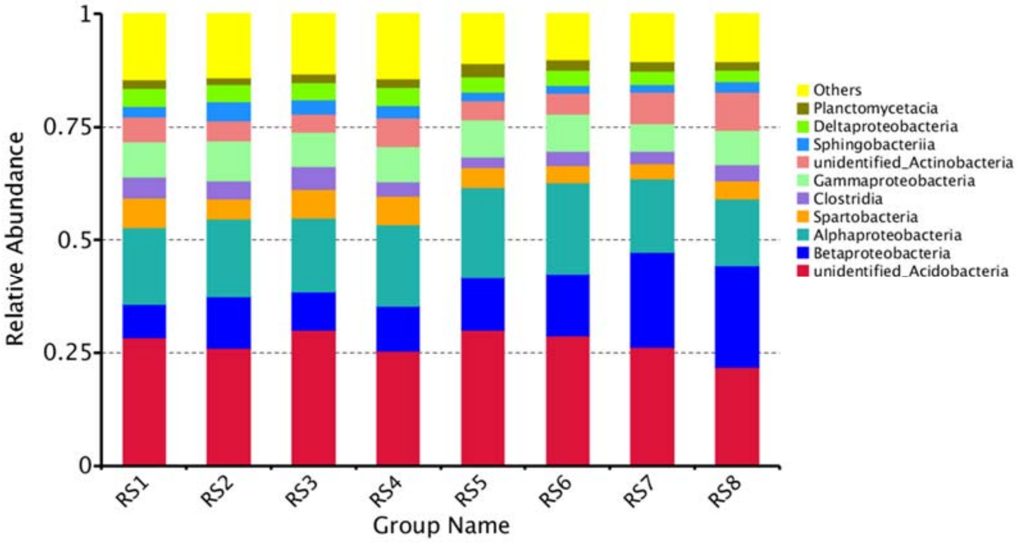

Supplement: Supplementary file 2 [file Data_Sheet_1.PDF]
